# Supplementary material for: Multi-wavelength anomalous diffraction de novo phasing using a two-colour X-ray free-electron laser with wide tunability
Source: Nat Commun. 2017 Oct 27;8:1170. doi: 10.1038/s41467-017-00754-7 (PMC5660077; doi:10.1038/s41467-017-00754-7)
Supplement: Supplementary file 1 — Supplementary Information Supplementary figures, supplementary table, supplementary notes, supplementary methods and supplementary references [file 41467_2017_754_MOESM1_ESM.pdf]

Description of Supplementary Files

File name: Supplementary Information
Description: Supplementary figures, supplementary table, supplementary notes, supplementary methods and supplementary references.

File name: Peer review file

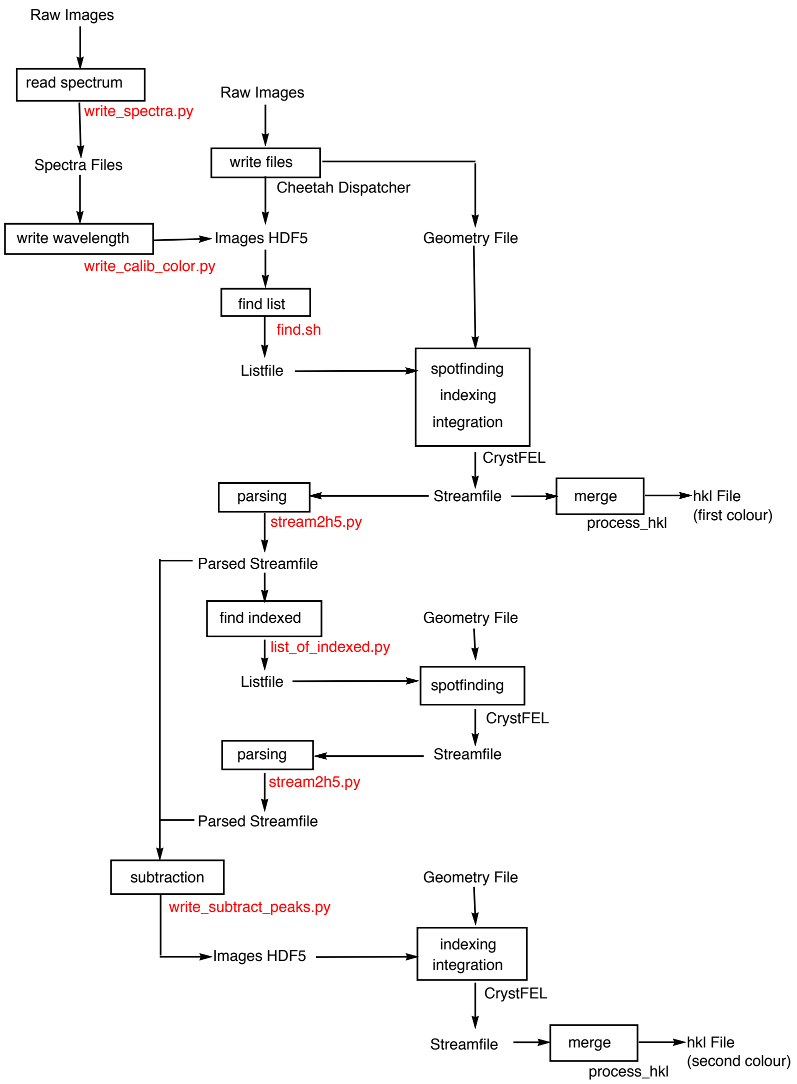

Supplementary Figure 1| Data analysis flowchart for two-colour SFX data. HDF5 hit images are retained by the Cheetah Dispatcher. The accurate wavelengths are added to the HDF5 images by the write_spectra.py and write_calib_color.py modules. Spotfinding, indexing and integration are performed for the dominant colour with the indexamajig tool from CrystFEL. All successfully indexed images are selected for the second processing round using the list_of_indexed.py module. indexamajig is used to obtain larger sets of diffraction peaks (by application of a lower threshold and signal-to-noise ratio). From these peaklists the peaks of the dominant colour are subtracted using write_subtract_peaks.py. Subsequently, the weak diffraction patterns are found, indexed and integrated. (Newly implemented modules are coloured in red.)

Supplementary Methods

Identification of high intensity spots using the interest point algorithm

Potential Bragg reflections are typically identified in a diffraction pattern by first recognizing high intensity spots by thresholding and the requirement to surpass a certain signal-to-noise ratio (snr). This can be challenging for strongly fluctuating or structured backgrounds and/or weak diffraction intensities of the spots. We encountered both issues. Therefore, we implemented an interest point algorithm[1]. The peak positions identified by this algorithm are referred to as interest points. These are pixels with certain properties such as absolute intensity value, absolute gradient value, mean neighboring intensity etc., that are characteristic for an image; typically they are used in computer vision algorithms.

The algorithm is based on a principal component analysis (PCA) dimension reduction technique from the multivariate data analysis[2,3].This technique identifies a n-dimensional projection vector along which the total variance of the n-dimensional data set is maximized. Thus along this vector individual data points can be distinguished most easily. By principle component analysis this algorithm determines automatically how to weight the set of n characteristics describing a pixel in order to decide whether it could be the centroid of a diffraction peak. Thus for each CCD module of the MPCCD detector (or any other detector) the most intense *num_peaks* are returned. As pixel characteristics we chose the Intensity Over Background Value (IOBV) and its Intensity Divided by the Background Value (IDBV). The set of characteristics can be expanded for example by the image moments[4] of the surrounding pixels which would be an estimate of the diffraction peak area.

The calculation of diffraction peak candidates is performed as follows:

- **Calculation of pixel characteristics**

  A background image was calculated by applying a moving median filter of a 11 pixel by 11 pixel window on the diffraction image.

  As the pixel area of the diffraction spots varies between 2 pixel^2 to 7 pixel^2 the size of the moving window was chosen such that the spot area is less than 6% of the number of pixels (121) in the window. When the median of the 121 pixels intensities is calculated, the high intensity of the spot appears to be an outlier in the intensity distribution. Therefore, it is discarded and does not contribute to the background estimate. This way, a robust background value was obtained for each pixel.

  A bias (i.e. a constant offset) was added to the diffraction image and the background image in order to normalize the minimal pixel read to a value of 100 to prevent division by 0 when the IDBV characteristic is calculated. Since the image contains a small fraction of negative pixel reads after the pedestals removal during image correction, the 0.9999 smallest value was selected as a threshold. Every pixel read below this value was set to 0 and then a value of 100 was added to each pixel.

For each pixel the IOBV and IDBV characteristics were calculated to form a set of 524288 (i.e. 1024*512) tuples.

This procedure was performed separaely for each of the 8 CCD modules of the detector.

- **Calculation of the principal component**

  For each pixel of the CCD module numerated by the index i the standardized feature vector ϕ_i^{standard} is calculated by subtracting the mean μ^{IOBV} respectively μ^{IBDV} and dividing by the standard deviation σ^{IODV} respectively σ^{IBDV}. This procedure is called "whitening" and is essential to find the principal components.

$$\text{IOBV}_i = I_i - B_i \tag{1}$$

$$\text{IBDV}_i = \frac{I_i}{B_i} \tag{2}$$

$$\phi_i = (\text{IOBV}_i, \text{IBDV}_i) \tag{3}$$

$$\phi_i^{\text{standard}} = \left(\frac{\text{IOBV}_i - \mu^{\text{IOBV}}}{\sigma^{\text{IOBV}}}, \frac{\text{IBDV}_i - \mu^{\text{IBDV}}}{\sigma^{\text{IBDV}}} \right) = \left(\text{IOBV}_i^{\text{standard}}, \text{IBDV}_i^{\text{standard}} \right) \tag{4}$$

From the set of standardized feature vectors ϕ_i^{standard} the covariance matrix is calculated Σ^{standard} and the eigenvectors v^C and eigenvalues λ^C are obtained:

$$C^{\text{standard}} = \text{Cov}\left(\text{IOBV}^{\text{standard}}, \text{IBDV}^{\text{standard}} \right) \tag{5}$$

$$= \frac{1}{k} \left(\sum_{i=1,,k} \left(\phi_i^{\text{standard}} \otimes \phi_i^{\text{standard}} \right) \right) \tag{6}$$

$$C^{\text{standard}} = \begin{pmatrix} C_{11} & C_{12} \\ C_{21} & C_{22} \end{pmatrix} \in \mathbf{R}^{2\times 2} \tag{7}$$

$$\text{Eig}\left(C^{\text{standard}} \right) = \left\{ \left(v_1^C, \lambda_1^C \right), \left(v_2^C, \lambda_2^C \right) \right\} \tag{8}$$

The eigenvector v_{max}^C with the largest eigenvalue λ_{max}^C is selected.

$$v_{\text{PCA}}^C = \begin{cases} v_{\text{max}}^C & v_{\text{max}}^C . \{1,1\} > 0 \\ -v_{\text{max}}^C & v_{\text{max}}^C . \{1,1\} < 0 \end{cases} \tag{9}$$

$$\text{score}_i = v_{\text{PCA}}^C . \phi_i^{\text{standard}} \tag{10}$$

A score value $score_i$ is assigned to each pixel to measure the likelihood of this pixel being a peak in the image. The principal component v_{PCA}^C needs to be oriented such that its projection on the vector $\{1,1\}$ is positive to ensure that if the score value increases the likelihood for being a peak increases as well.

- **Selecting the *num_peaks* best pixel candidates**

  Each pixel i is sorted by its score $score_i$ in descending order into a list to ensure that pixels with high likelihood of being a peak have a lower list index l.

  To ensure that *num_peaks* centroid positions of distinct peaks are returned by this algorithm, an exclusion zone is set to prevent that two peak positions are closer than 10 pixels to each other. A selection algorithm chooses the first *num_peaks* pixels that are not within 10 pixels of each other and returns their positions as output to the HDF5 image file.

  This process is repeated for each CCD module of the MPCCD detector.

The write_pca_peaks.py module is implemented in the Python programming language and makes use of the SciPy library. After calculating the user-specified number of peaks per CCD module their positions are written into the HDF5-format image file. Thus when the image is processed by CrystFEL's indexamajig module[5] these positions are available and the peak detection step can be bypassed.

In conclusion, the write_pca_peaks.py algorithm was developed and implemented as a standalone module to replace the peak detection step implemented in the indexamajig module in CrystFEL that relies on thresholding, requiring a number of user-supplied parameters (threshold, signal-to-noise ratio and minimal gradient). Instead, write_pca_peaks.py performs automatic peak parameter selection for each individual diffraction image. It requires a detector geometry file parsed by the stream2h5.py module (geometry.h5), the path of a diffraction image file in HDF5 fileformat, and the user-specified number of peaks to be found for each CCD module (*num_peaks* parameter) as input and returns the specified number of good candidate peak positions that can then be used for indexing. We used the write_pca_peaks.py algorithm to extract the positions of Debye-Scherrer rings in powder patterns for the sample-to-detector distance calculation. It can also be used for the two-colour diffraction dataset replacing the peak detection step performed by CrystFEL's indexamajig module[5].

Distance parameter calculation

Typically, the sample-to-detector distance is not known accurately at the beginning of a SFX experiment. Moreover, it can change between different experiments or during the experiment itself for various reasons. Thus, before processing a crystallographic dataset the detector distance parameter needs to be determined accurately.

Processing of the two-colour diffraction dataset with the provided detector distance ($51.5\,mm$) performed poorly during the indexing step which rendered an accurate experimental determination of the detector distance mandatory. To this end, a silicon powder diffraction dataset was recorded. Each of the recorded diffraction patterns contained three visible Debye-Scherrer rings. With the tabulated values of the diffraction plane spacings of silicon crystals[6] the detector distance can be calculated using Bragg's law and the projection equation.

In order to obtain all parameters required for distance calculation the Debye-Scherrer ring radii needed to be extracted from the powder images, associated

with the corresponding Miller indices of the diffracting planes of the silicon crystals and assigned the respective wavelength of the colour producing the diffraction pattern.

Extracting the powder ring points

In the central parts of the detector the strong background signal from the grease carrier medium containing the silicon nanocrystals by far exceeded the intensity of the outer silicon Debye-Scherrer rings. This made selecting a uniform threshold impossible; thus these signals could not be separated from the background and from each other by a threshold method. To solve this problem and to extract the positions of pixels being likely part of the diffraction ring pattern the interest point algorithm was applied.

300 interest points were calculated for each CCD module (in total 2400) to ensure that the obtained set of points would contain the diffraction rings as subsets. By a K-Means clustering[3] of the radial distances (of the individual points) the set of interest points could be subdivided into three sets containing only points from one ring manifold. For each of these subsets the radial point density was calculated by a kernel density estimation method[3] and the most likely radius was chosen (modus). Thus from each image containing a silicon powder pattern three estimates for the ring radii were obtained.

Calculations based on approximate values for detector distance and X-ray wavelength led to the conclusion that the observed ring patterns were produced by the 9keV colour.

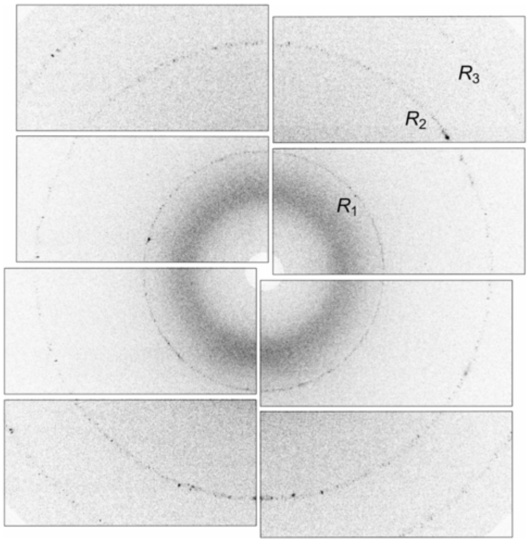

Supplementary Figure 2| Silicon powder pattern recorded with the MPCCD detector for the sample-to-detector distance determination. For purposes of presentation the colour was inverted such that black represents high intensity pixel values and vice versa. The photons of one of the two colours of the X-ray beam are scattered by randomly oriented silicon nanocrystals at characteristic angles into three rings (R_1, R_2, R_3); the diffraction by the second colour is extremely weak. The broad diffuse ring is produced by scattering from the random ordered grease molecules of the carrier medium.

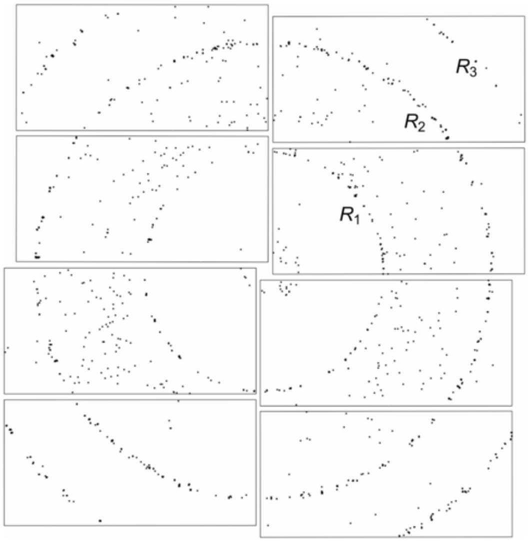

Supplementary Figure 3| Plot of points extracted from the powder image using the interest point algorithm. 300 points with the highest dynamically calculated scores were selected for each detector CCD module. The identified points indicate not only diffraction rings but additional rings originating from higher X-ray harmonics and possibly crystalline chemical additives in the grease carrier medium.

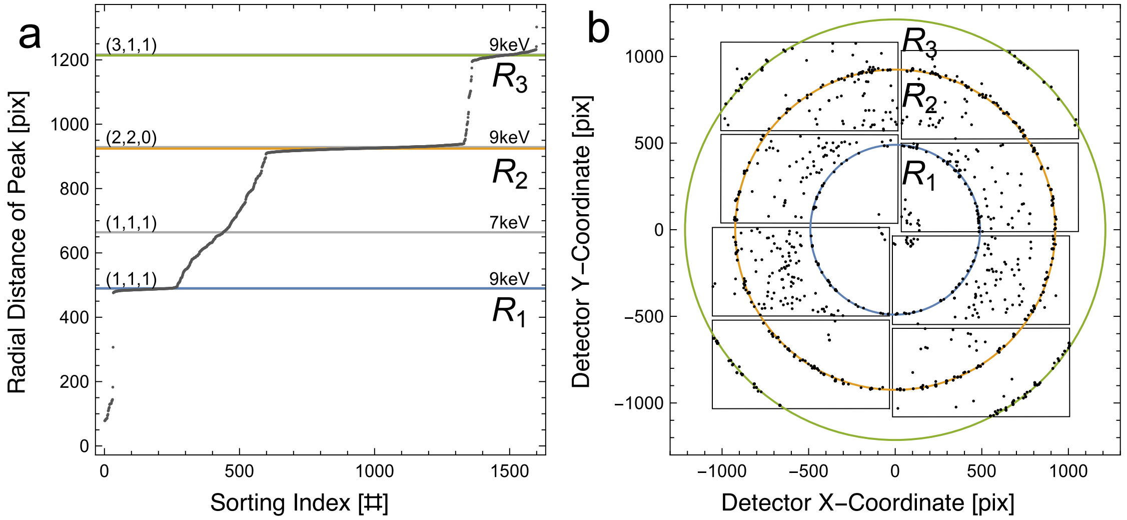

Supplementary Figure 4| Identification of Debye-Scherrer rings. (**a**) K-means clustering into ring manifolds. Using Bragg's law and the tabulated values for the diffraction plane spacings[6] the wavelength associated with the (**b**) Debye-Scherrer rings could be identified (assuming a detector distance of $d = 51.5\,mm$).

Supplementary Table 1| Expected and measured ring radii for Debye Scherrer rings of the silicon powder calculated for $d = 51.5\,mm$. Values were obtained from the American Mineralogist Crystal Structure Database[6].

Miller index	Ring Name	Spacing	Radius 7keV	Radius 9keV	Measured Radius 9keV
		[Å]	[pixel]	[pixel]	[pixel]
(1,1,1)	R_1	3.1357	664.04	488.61	484.9 $\pm$ 0.86
(2,2,0)	R_2	1.9202	1467.14	928.79	920.58 $\pm$ 2.34
(3,1,1)	R_3	1.6376	2257.52	1216.55	1209.39 $\pm$ 3.36

Distance estimation without wavelength parameter

For the powder diffraction images the exact wavelength value for the ∼9keV colour were not available. Thus the distance calculation needed to be performed without a precise wavelength value and an algorithm had to be developed for this purpose. Each diffraction image contained multiple powder rings produced by the same colour. From these powder-rings the ring-radii r_i were obtained. The associated lattice spacings d_i of these rings are known.

As derived in the next section, using Bragg's law and the projection equation the following equation can be obtained:

$$\frac{1 \pm \frac{1}{\sqrt{\left(\frac{r_1}{D}\right)^2+1}}}{1 \pm \frac{1}{\sqrt{\left(\frac{r_2}{D}\right)^2+1}}} = \left(\frac{d_2}{d_1}\right)^2 \tag{11}$$

This equation contains only the detector distance (D), the ring radii (r_1, r_2) and the lattice spacings (d_1, d_2) as variables. In principle, this equation can be solved for the detector distance D but in practice, because of the extreme algebraic effort, this is not feasible.

Instead, a numerical method for equation-solving was used to obtain a sample-to-detector distance value that is more accurate than the default. For this purpose a score function (error function) measuring the equality of the terms on the left and the right hand side of equation (11) was defined. It was used to estimate the quality of a value for the sample-to-detector distance given the observed ring radii and the associated lattice spacings.

Derivation of the score function for detector distance estimation by optimization

Starting with Bragg's law and the projection equation:

$$2\,d_i \sin(\theta_i) = n\,\lambda \tag{12}$$

$$\tan(2\,\theta_i) = \frac{r_i}{D} \tag{13}$$

where: d_i : spacing of diffracting sub lattice

θ_i : diffraction angle

λ : wavelength of diffracted light

r_i : radius of diffraction powder ring

D : distance to the detector

$$
\begin{aligned}
\tan\left(2\,\theta_i\right) &= \\
\frac{\sin(2\,\theta_i)}{\cos(2\,\theta_i)} &= \\
\frac{\sqrt{1-\cos^2(2\,\theta_i)}}{\cos(2\,\theta_i)} &= \frac{r_i}{D} \\
\Leftrightarrow \qquad 1-\cos^2(2\,\theta_i) &= \left(\frac{r_i}{D}\right)^2 \cos^2(2\,\theta_i) \\
\Leftrightarrow \qquad \frac{1}{\left(\frac{r_i}{D}\right)^2+1} &= \cos^2(2\,\theta_i) \\
\Leftrightarrow \qquad 1-\frac{1}{\left(\frac{r_i}{D}\right)^2+1} &= \sin^2(2\,\theta_i) \\
&= \left(2\sin\left(\theta_i\right)\cos\left(\theta_i\right)\right)^2 \\
&= 4\sin^2\left(\theta_i\right)\cos^2\left(\theta_i\right) \\
&= 4\sin^2\left(\theta_i\right)\left(1-\sin^2\left(\theta_i\right)\right) \\
\Leftrightarrow \qquad \frac{1}{4}\left(\frac{1}{\left(\frac{r_i}{D}\right)^2+1}-1\right) &= \sin^4\left(\theta_i\right)-\sin^2\left(\theta_i\right) \\
\Leftrightarrow \qquad \frac{1}{4\left(\left(\frac{r_i}{D}\right)^2+1\right)} &= \left(\sin^2\left(\theta_i\right)-\frac{1}{2}\right)^2 \\
\Leftrightarrow \qquad \pm\frac{1}{2}\sqrt{\frac{1}{\left(\frac{r_i}{D}\right)^2+1}} &= \sin^2\left(\theta_i\right)-\frac{1}{2} \\
\Leftrightarrow \qquad \frac{1}{2}\left(1\pm\frac{1}{\sqrt{\left(\frac{r_i}{D}\right)^2+1}}\right) &= \sin^2\left(\theta_i\right)
\end{aligned}
$$

$$
\begin{aligned}
\Leftrightarrow \qquad \pm\sqrt{\frac{1}{2}\left(1\pm\frac{1}{\sqrt{\left(\frac{r_i}{D}\right)^2+1}}\right)} &= \sin\left(\theta_i\right) \\
&= \frac{\lambda}{2d_i}
\end{aligned}
$$

We arrive at a function which only depends on the detector distance, lattice spacings and powder ring radii.

$$
\Rightarrow \qquad \frac{1\pm\frac{1}{\sqrt{\left(\frac{r_1}{D}\right)^2+1}}}{1\pm\frac{1}{\sqrt{\left(\frac{r_2}{D}\right)^2+1}}} = \left(\frac{d_2}{d_1}\right)^2 \tag{14}
$$

From this equation two possible score functions can be defined:

$$score_{(+)}[D|r_1, r_2, d_1, d_2] = \left\| \frac{1 + \frac{1}{\sqrt{\left(\frac{\mathrm{r1}}{D}\right)^2 + 1}}}{1 + \frac{1}{\sqrt{\left(\frac{\mathrm{r2}}{D}\right)^2 + 1}}} - \left(\frac{d_2}{d_1}\right)^2 \right\| \quad (15)$$

$$score_{(-)}[D|r_1, r_2, d_1, d_2] = \left\| \frac{1 - \frac{1}{\sqrt{\left(\frac{\mathrm{r1}}{D}\right)^2 + 1}}}{1 - \frac{1}{\sqrt{\left(\frac{\mathrm{r2}}{D}\right)^2 + 1}}} - \left(\frac{d_2}{d_1}\right)^2 \right\| \quad (16)$$

$$D_{\mathrm{opt}} = argmin_{D \in \{D_{\min}, D_{\max}\}}[score[D|r_1, r_2, d_1, d_2]] \quad (17)$$

where: $score$: score function for the optimal distance estimate
D : distance between the interaction zone and the detector
r_i : radius of observed ring pattern
d_i : spacing of diffracting sublattice

Only the score$_{(-)}$ function is suited for the numerical equation-solving method as is apparent from the plot of the score functions shown in Supplementary Figure 5.

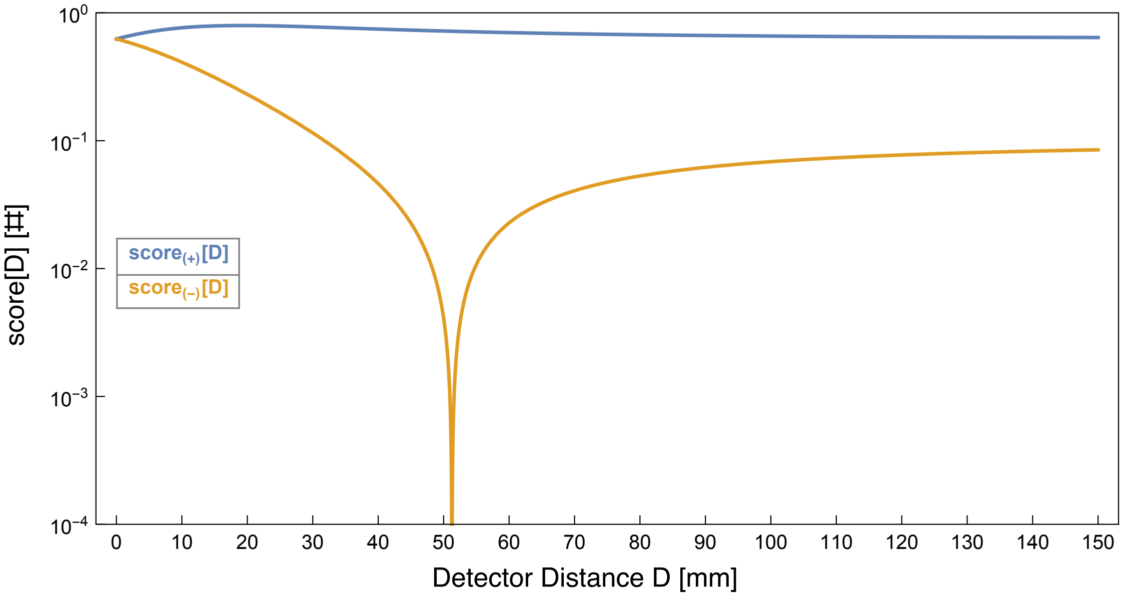

Supplementary Figure 5| Plot of the score function for detector distance optimization. Plots of the score$_{(+)}$ and the score$_{(-)}$ functions. The score$_{(-)}$ function (orange) is convex and has one global minimum, while the score$_{(+)}$ function (blue) is concave and has its local extrema in 0 and ∞ which are improper values for the detector distance. Thus the score$_{(+)}$ function is not applicable for finding the detector distance by minimizing the score.

Distance parameter calculation

By a numerical minimization of the $\text{score}_{(-)}$ function starting at the default distance value of 51.5mm a better value for the detector distance was found; it had the smallest score and thus satisfied equality (11). Each detector distance computation requires four parameters to be known (r_1, r_2, d_1, d_2). Six independent parameter values can be obtained (i.e. three radii r_i and their associated lattice spacings d_i) from the recorded silicon powder pattern produced by the 9keV colour. Thus three independent detector distance estimates were determined from one powder pattern image. The probability density for the sample-to-detector distance parameter (obtained using kernel density estimation) is shown in Supplementary Figure 6.

The median of the computed sample-to-detector distance values $\mu_{\text{med}} = 51.03\text{mm}$ was chosen as the best detector distance value and used in further data processing steps.

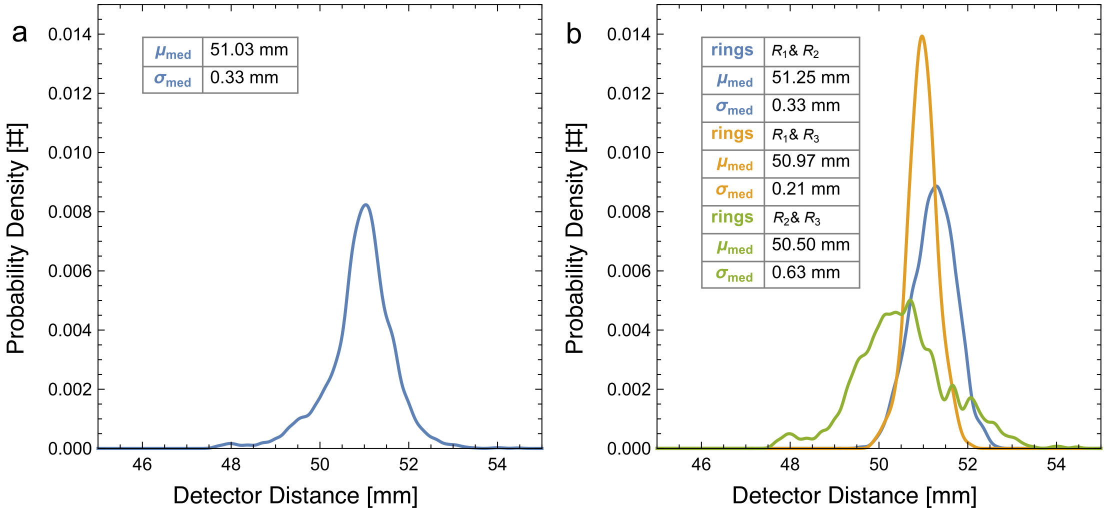

Supplementary Figure 6| Plot of the probability density of the detector distance estimates. (**a**) Plot of the probability density obtained by kernel density estimation from the total set of computed detector distances. (**b**) Plot of the probability density obtained by kernel density estimation from the detector distances that were computed with ring parameters (i.e. radius r_i and the associated spacing d_i) of two selected powder rings (R_1 inner powder ring, R_2 middle powder ring, R_3 outer powder ring). For the three combinations of ring pairs three different probability functions are obtained. When ring parameters for R_1 (inner ring) and R_3 (outer ring) are chosen for computation of the detector distance a narrow probability density is obtained.

Supplementary Note 1

Calibration of the wide-range inline spectrometer

The energy calibration of the wide-range inline spectrometer was obtained from the comparison between the readings of the wide-range and the narrow-range inline spectrometers. In two reference runs the respective photon energy (7 keV or 9 keV) was probed by the narrow-range inline spectrometer. Since the resolving power of the narrow-range spectrometer is lower, for some different readings of the wide-range spectrometer the narrow-range spectrometer displays the same energy. By calculating the median of the wide-range spectrometer readings that have an identical narrow-range spectrometer value, points are obtained that are then used for the estimation of the calibration function. A linear model fit was performed with these points. The final calibration functions are shown in Supplementary Figures 10 and 11.

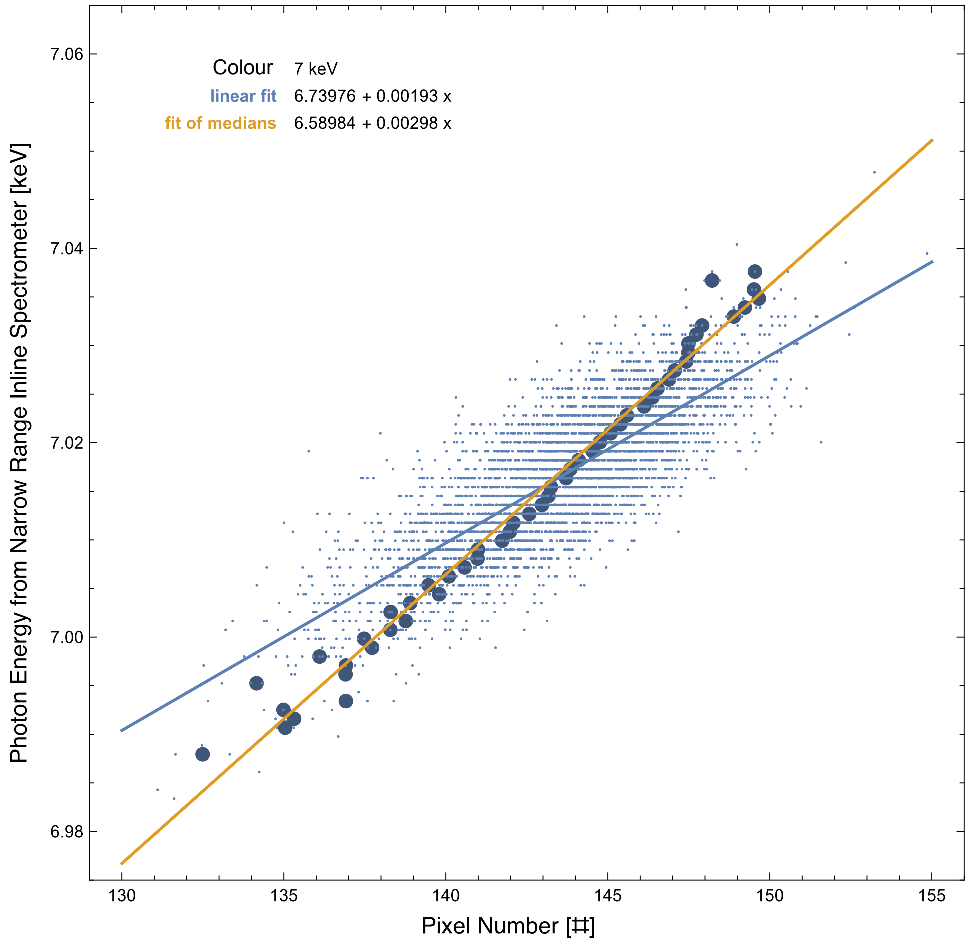

Supplementary Figure 7| The calibration functions obtained from linear model fit and from fit of medians (thick blue spheres) for the 7keV colour. The calibration data cannot be explained by a linear model due to the present stripe pattern.

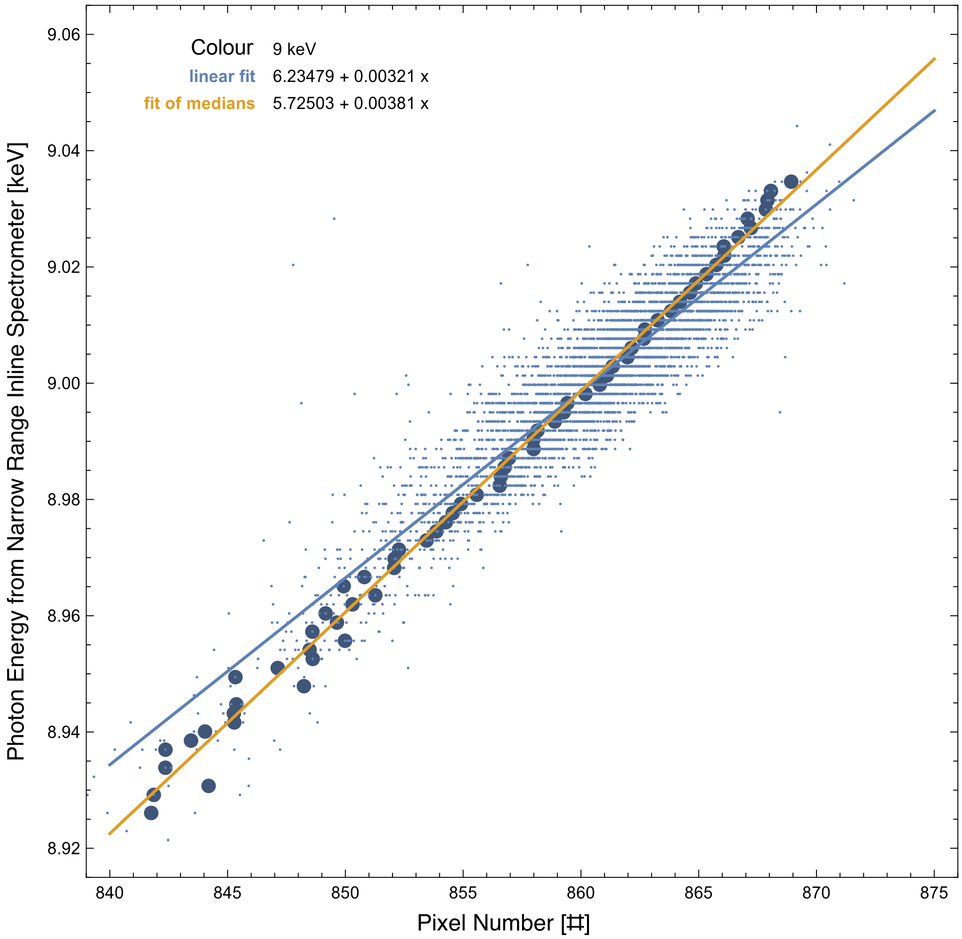

Supplementary Figure 8| The calibration functions obtained from linear model fit and from fit of medians (thick blue spheres) for the 9keV colour. The calibration data cannot be explained by a linear model due to the present stripe pattern.

Supplementary Note 2

Processing the two-colour data

Once the final processing parameters were chosen and each of the diffraction images was processed for each of the two colours with CrystFEL's indexamajig module it turned out that only an extremely small fraction of images could be indexed in two colours. Therefore, a new strategy was developed to separate the spots of the two diffraction patterns. This procedure was used successfully to index a large fraction of the diffraction patterns in two colours.

Indexing the dominant diffraction pattern

The diffraction images of the two-colour data set contain two different diffraction patterns that need to be processed separately. In general, in our experiment one of the two colours was brighter than the other. Therefore the diffraction pattern of one of the two colours dominates and prevents the direct processing of the complementary colour, as the diffraction spots from the brighter colour are more likely to be detected and used in the image processing. Thus, more diffraction spots from the first colour are used for the calculation of the lattice basis. The combination of these spots with the wavelength of the second colour results in the prediction of lattice parameter that are inconsistent with the ones predicted using the first colour, and the image will be discarded as not indexable since it does not fit the chosen cell parameters.

In the first step each of the diffraction images was regularly processed with the following parameters and against the unit cell parameters to discard indexing results with different lattice types.

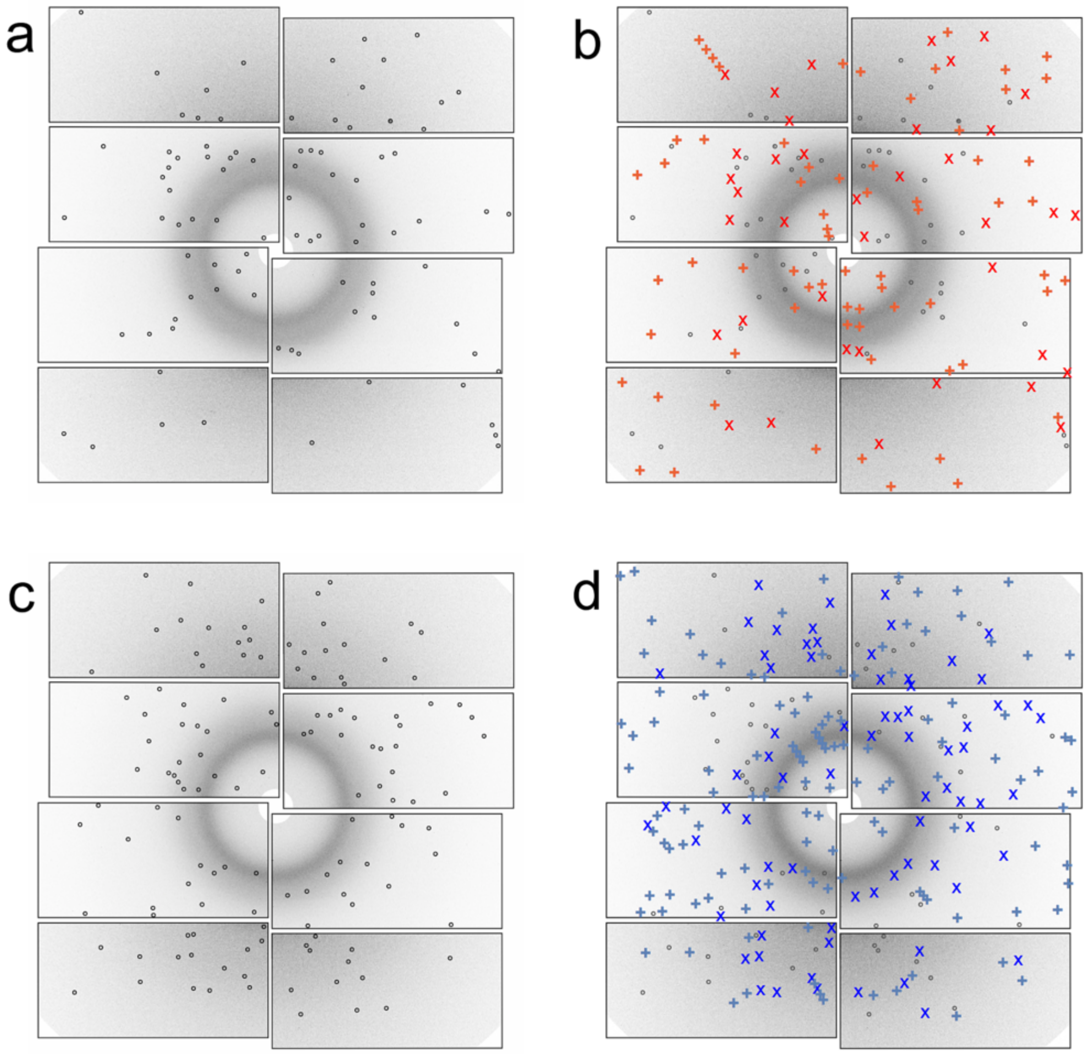

Supplementary Figure 9| Illustration of the first processing step of the diffraction image processing.

(**a**) The identified peaks are plotted at the position where they were found in the diffraction image. Image (**a**) is indexable in the 7keV colour but not directly in the 9keV colour and for image (**c**) it is reversed. When these images are processed with the respective colour information most of the identified peaks could be used to calculate the reciprocal lattice basis and indexed. These points are marked by the X in the images (**b**) and (**d**). CrystFEL's indexamajig module predicts on the basis of some beam parameter settings and the calculated lattice basis other possibly observable diffraction spots. Their positions are marked as + and were not detected as peaks in the diffraction image because they either are not present or too weak to be detected by the peak search algorithm given the set parameters. Those peaks which could not be explained by the predictions are marked as circles. (The processing parameters for indexamajig were: median-filter 16, threshold 200, min-snr 5, min-gradient 10000, tolerance 10,10,2 and int-radius 6,6,8.)

Finding the weak diffraction pattern

To find the second diffraction pattern in the diffraction image the peak search parameters were lowered to have a larger set of peaks to search for the diffraction pattern. As some of the peaks identified in the second step are part of the dominant diffraction pattern they need to be discarded from the search.

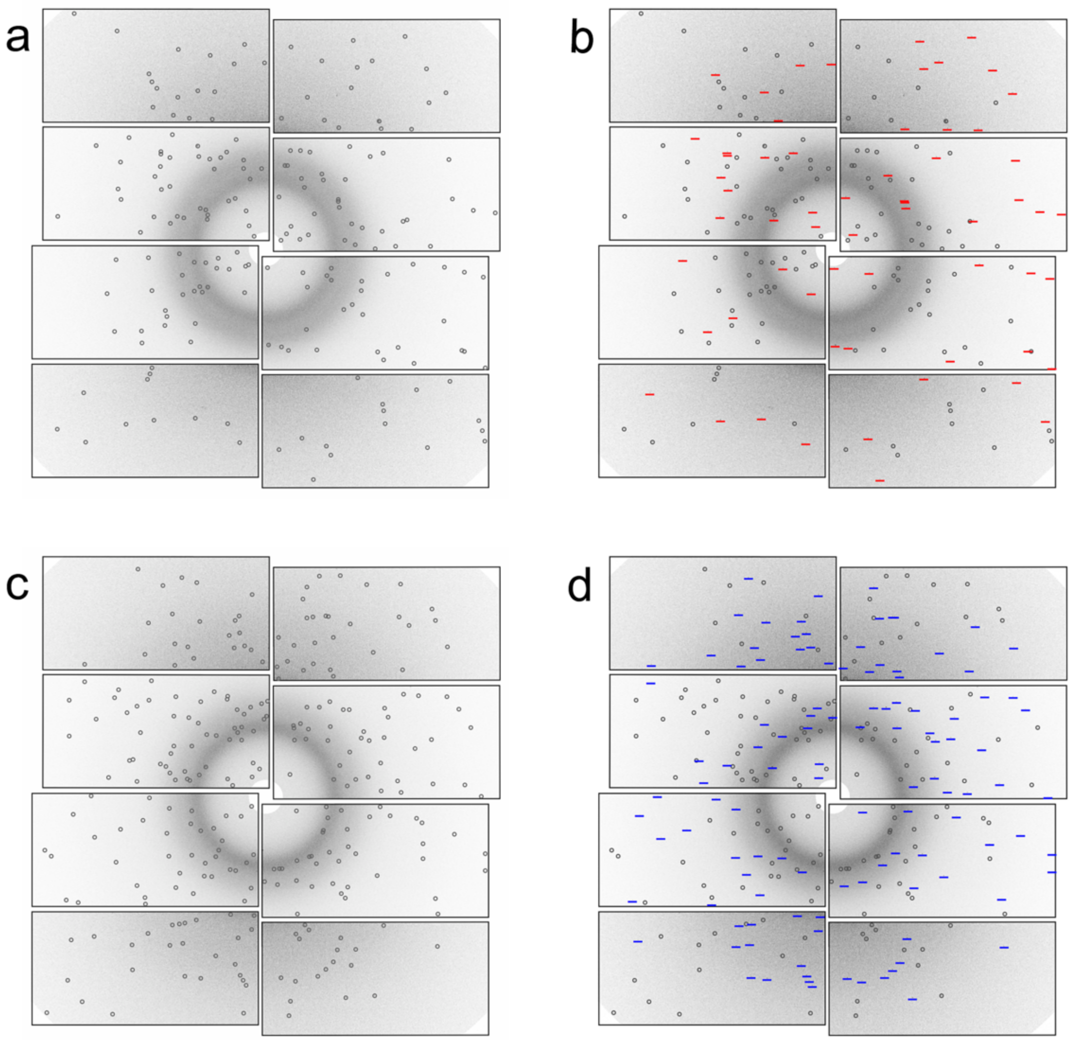

Supplementary Figure 10| Illustration of the procedure to discover likely diffraction peaks of the second diffraction pattern. As the exemplary image (**a**) is only directly indexable in the 7keV colour and image (**c**) in the 9keV colour a different set of parameters was used to find more low intensity peaks in the image. All found peaks which were in the vicinity of diffraction spots within 10 pixels were discarded. These peaks are marked by the – symbol in image (**b**) and (**d**). The remaining set of peaks was used for data processing for the 9keV colour. (The processing parameters for indexamajig were: median-filter 16, threshold 150, min-snr 3, min-gradient 10000, tolerance 10,10,10,2, int-radius 6,6,8).

Reprocessing with low intensity diffraction peaks

The positions of the low intensity peaks, obtained in the second step, were passed to CrystFEL's indexamajig module to perform the processing of the diffraction images and to skip the peak detection step. When the positions of peaks are provided to indexamajig the peak detection parameters are ignored.

The reciprocal lattice basis is calculated from the positions of these peaks and is then used to predict the diffraction pattern. As the cell parameters were provided in the reprocessing step this additional information greatly increased the indexing rate since the processing tool can discard implausible peaks.

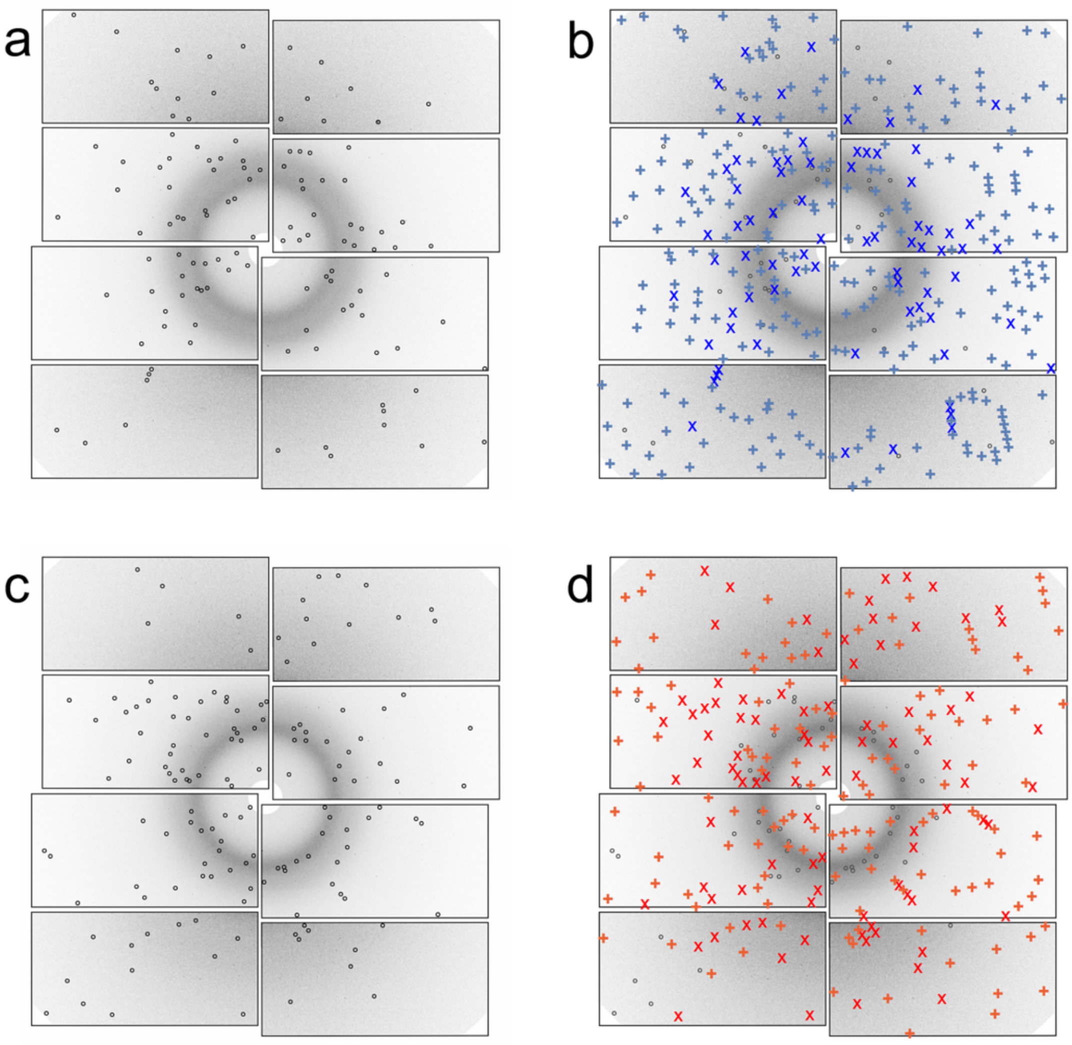

Supplementary Figure 11| Illustration of the third reprocessing step. In image (**a**) and (**c**) the low intensity peaks are plotted that do not coincide with the diffraction spots of the primary diffraction pattern. In image (**b**) and (**d**) the predicted secondary diffraction pattern is plotted. Peaks that could be indexed are marked as `x` while newly predicted peaks are marked as `+`. The residual peaks are indicated by circles. Thus image (**a**) which could not be indexed in the 9keV colour before was indexed and processed after the additional procedure.

Supplementary References

1. Schmid, C., Mohr, R. and Bauckhage, C. Evaluation of Interest Point Detectors. *International Journal of Computer Vision*, **37**,151 - 172 (2000).

2. Pearson, K. On Lines and Planes of Closest Fit to Systems of Points in Space. *Philosophical Magazine*, **2**,559 - 572 (1901).

3. Hastie, T., Tibshirani, R., and Friedman, J. (Second edition 2006).
   The Elements of Statistical Learning Data Mining and Inference and and Prediction.
   Springer.

4. Hu, M.K. Visual Pattern Recognition by Moment Invariants.
   IRE Trans.Info.Theory, **8**,179 - 187 (1962).

5. White, T. A., Kirian, R. A., Martin, A. V., Aquila, A., Nass, K., Barty, A., and Chapman., H. N. CrystFEL: a software suite for snapshot serial crystallography. *J. Appl. Cryst.*, **45**,335 - 341 (2012).

6. Downs, R.T. and Hall-Wallace, M.
   The American Mineralogist Crystal Structure Database.
   American Mineralogist **88**, 247 - 250 (2003)
